# Activation of Dinitrogen as A Dipolarophile in 1,3-Dipolar Cycloadditions: A Theoretical Study Using Nitrile Imines as “Octet” 1,3-Dipoles

**DOI:** 10.1038/s41598-017-05708-z

**Published:** 2017-07-21

**Authors:** M. Merced Montero-Campillo, Ibon Alkorta, José Elguero

**Affiliations:** Instituto de Química Médica, CSIC, Juan de la Cierva, 3, E-28006 Madrid, Spain

## Abstract

Theoretical calculations at the G4MP2 level of theory demonstrate that it is possible to activate dinitrogen to make it react in dipolar cycloadditions using neutral beryllium derivatives and other neutral metallic compounds. For the particular case of beryllium, the barrier decreases more than 40 kJ·mol^–1^ with respect to the non-catalysed reaction. The activation achieved is lower than using diazonium salts (models of protonated N_2_), but still in a range that can be experimentally attainable.

## Introduction

**Figure 1:**
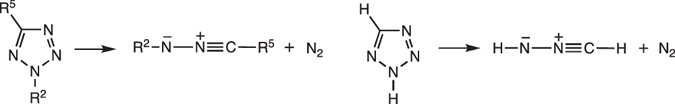
Ring opening of N_2_-substituted tetrazoles.

It is well known that dinitrogen, N_2_, does not react with 1,3-dipoles, a fact that has been attributed to a very large HOMO-LUMO gap^1^. However, the backward reaction, 2,5-disubstituted tetrazoles yielding nitrile imines^2,3^ plus dinitrogen, was reported by Huisgen in 1959 (Fig. 1)^4,5^. Using a simplified model (R^2^ = R^5^ = H), the ring-opening reaction was studied theoretically by Kiselev *et al*.^6^ which reported barriers of 153.6 (Δ*G*) and 159.4 kJ·mol^–1^ (Δ*H*).

Concerning this question, there are only two references reporting experimental results. Fürstner *et al*. described that the {N_2_} unit in aryldiazonium salts undergoes facile triple-bond metathesis using Mo or W complexes; they explain this by a polarization of the {N≡N} unit^7^. Ma *et al*. described the facile reaction of arene-diazonium salts with 2,2,2-trifluorodiazoethane to yield 2-aryl-5-trifluoromethyltetrazoles, but the reaction needs silver as a catalyst and the mechanism is not disclosed^8^.

We decided to explore if the coordination of N_2_ with several neutral molecules is able to lower the barrier to reasonable values. With this goal in mind, we related it with the ability of BeH_2_ to form stable complexes with N_2_^9^. As a reference, we chose the 1,3-dipolar cycloaddition between dimethyl nitrile imine and dimethyl acetylene, a reaction that proceeds smoothly in the case of diphenyl nitrile imine^3,5,10^.

## Results and Discussion

**Figure 2:**
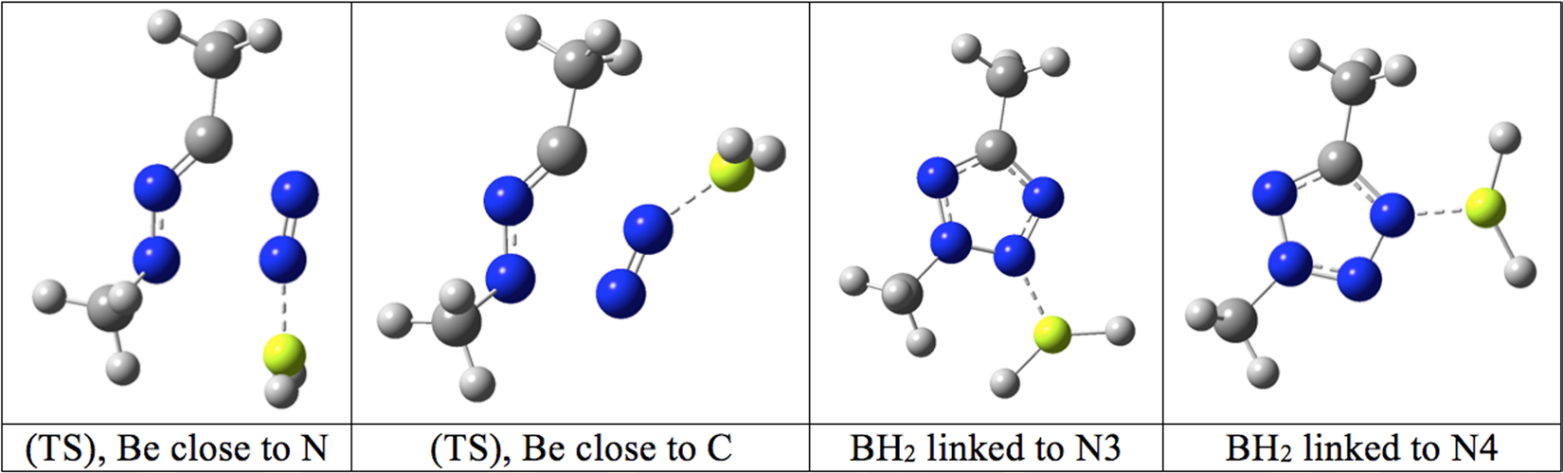
Transition states and products of the reaction between H_2_Be···N_2_ and dimethyl nitrile imine at the G4MP2 level of theory.

**Figure 3:**
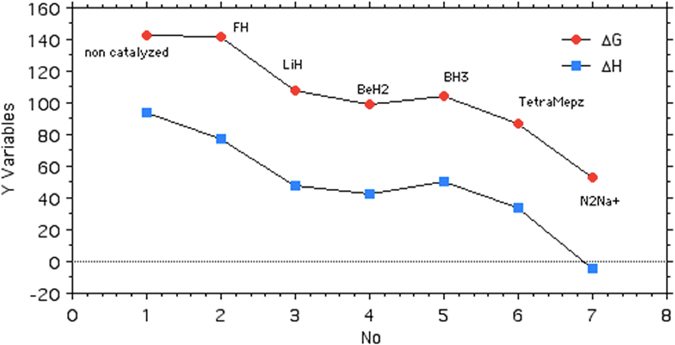
Energy profiles [Y Variables, Δ*G* (red) and Δ*H* (blue)] in kJ·mol^−1^ corresponding to the barriers shown in Table 2.

We report in Table 1 the results obtained at the G4MP2 theoretical level (see Methods Section). Results include the non-catalysed reaction (N_2_ + dimethyl nitrile imine), the activation of N_2_ by a hydrogen bond (HF), by metals (Li, Be, B) in their corresponding hydride forms, and the abovementioned reference reaction^11^. Figure 2 exemplifies the Be case. Table 1 also includes the same dipolar cycloaddition using N_2_Na^+^ as a dipolarophile, a case that will be explained later on. Table 2 summarizes the lowest barriers for each studied reaction, the enthalpy and free energy contributions being depicted in Fig. 3. For the ring opening of 2,5-dimethyltetrazol, we find barriers of 167.4 (Δ*G* = 142.5 + 24.9) and 171 kJ·mol^–1^ (Δ*H* = 93.9 + 77.2), values that compare well with those reported for the non-substituted derivative, 153.6 (Δ*G*) and 159.4 kJ·mol^–1^ (Δ*H*)^6^.

**Table 1:** Enthalpy, free energy and entropy contributions (kJ·mol^–1^) for the 1,3-dipolar cycloaddition between dimethyl nitrile imine and different dipolarophiles at the G4MP2 level (T = 298.15 K).

Tetrazoles	∆*H*	∆*G*	−T∆*S*
**Reagents: N**_**2**_	0.0	0.0	
2,5-Dimethyltetrazol (TS)	93.9	142.5	48.6
2,5-Dimethyltetrazol	−77.2	−24.9	52.3
**Reagents: N**_**2**_ **···HF**	0.0	0.0	
FH (TS), F close to NMe	77.7	141.5	63.8
FH (TS), F close to CMe	80.8	146.4	65.6
FH HB to N3	−105.2	−28.5	76.7
FH HB to N4	−108.4	−37.0	71.4
**Reagents: N**_**2**_ **···LiH**	0.0	0.0	
LiH (TS), Li close to N	48.1	107.5	59.4
LiH (TS), Li close to C	56.1	115.7	59.6
LiH linked to N3	−138.0	−74.8	63.2
LiH linked to N4	−139.6	−82.2	57.4
**Reagents: N**_**2**_ **···BeH**_**2**_	0.0	0.0	
BeH_2_ (TS), Be close to N	42.5	98.9	56.4
BeH_2_ (TS), Be close to C	46.5	105.4	58.9
BeH_2_ linked to N3	−151.4	−90.0	61.4
BeH_2_ linked to N4	−171.7	−109.2	62.5
**Reagents: N**_**2**_ **···BH**_**3**_	0.0	0.0	
BH_3_ (TS), B close to N	52.8	104.2	51.4
BH_3_ (TS), B close to C	50.4	104.0	53.6
BH_3_ linked to N3	−163.5	−104.5	59.0
BH_3_ linked to N4	−180.3	−120.6	59.7
**Reagents: N**_**2**_ **Na** ^**+**^	0.0	0.0	
TS, Na close to N	−4.1	52.9	57.0
Na linked to N3	−189.1	−131.1	58.1
**Pyrazoles**			
**Reagents: CH**_**3**_ **CCCH**_**3**_	0.0	0.0	
1,3,4,5-Tetramethylpyrazole (TS)	34.1	86.9	52.8
1,3,4,5-Tetramethylpyrazole	−425.1	−362.0	63.1

Dimethyl nitrile imine is omitted as a reagent for the sake of clarity.

**Table 2:** Lowest barriers (kJ·mol^−1^) of the set of 1,3-dipolar cycloadditions. Calculations were carried out at the G4MP2 level of theory for T = 298.15 K. See also Fig. 3.

No	∆*H*	∆*G*	−T∆*S*
**(1)** 2,5-Dimethyltetrazol (TS)	93.9	142.5	48.6
**(2)** FH (TS), F close to NMe	77.7	141.5	63.8
**(3)** LiH (TS), Li close to N	48.1	107.5	59.4
**(4)** BeH_2_ (TS), Be close to N	42.5	98.9	56.4
**(5)** BH_3_ (TS), B close to C	50.4	104.0	53.6
**(6)** 1,3,4,5-Tetramethylpyrazole (TS)	34.1	86.9	52.8
**(7)** NaN_2_^+^ (TS), Na close to N	−4.1	52.9	57.0

Remarkably, the coordination of BeH_2_ to N_2_ lowers the Δ*G* barrier from 142.5 to 98.9 kJ·mol^–1^, a value close to that of a CC triple bond (86.9 kJ·mol^–1^). Coordination of LiH and BH_3_ to N_2_ also has a significant effect on the barrier, whereas HF has almost no effect. Taking into account the already mentioned reactivity of diazonium salts, we decided to include an example in our survey. As a model of diazonium salts we first tried N_2_H^+^ (diazenylium, an interstellar molecule)^12^, but the very acidic proton is spontaneously transferred to the dipole. Then, we tried N_2_Na^+^ and obtained the barriers reported in Table 2, observing an extraordinary decrease of the activation energy.

In summary, while a hydrogen bond is not enough and diazonium is excellent but impractical, there exists a series of neutral molecules that are both practical and efficient, the best one being BeH_2_.

Additional calculations were carried out to analyse the reasons behind the strong activation by beryllium dihydride. The results provided by the analysis of the MESP (Molecular Electrostatic Potential^13^, see the Supporting Information file) indicate that a Lewis acid such as BeH_2_ should bind tetrazole stronger than the corresponding TS, being the complex formed with N_2_ much weaker. This could partially explain the stabilization of the TS and tetrazole.

**Figure 4:**
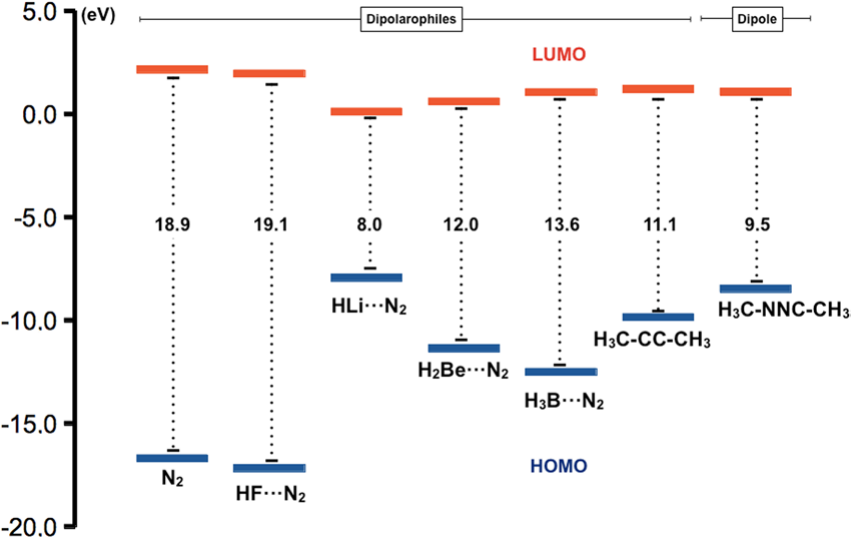
HOMO-LUMO gaps (eV) for the different neutral dipolarophiles (N_2_, FH···N_2_, HLi···N_2_, H_2_Be···N_2_, H_3_B···N_2_, H_3_C-CC-CH_3_) and the dipole (H_3_CNNCCH_3_) at the G4MP2 level of theory.

Going a step further, we carried out a Frontier Molecular Orbital (FMO) analysis of the different reactions. Reactivity in pericyclic reactions is usually explained by means of the FMO Theory^14^. The orbitals of the reagents in the ground state are used to predict the way the reaction is going to take place. According to Woodward-Hoffmann rules^15^, for a 4π + 2π dipolar reaction in thermal conditions as the one we are interested in, the symmetry-allowed pathway involves the supra-supra overlap of the frontier orbitals of the reagents (_4_π_s_ + _2_π_s_). There are two possible overlaps between the dipole and the dipolarophile: (i) The HOMO of the dipole + the LUMO of the dipolarophile; (ii) the LUMO of the dipole + the HOMO of the dipolarophile. The preferred pathway is predicted to be the one that implies the lowest gap between the dipole and the dipolarophile. In our particular case, the lowest gap is the one between the HOMO of the dipole and the LUMO of the dipolarophile, as expected for a nucleophilic dipole such as dimethyl nitrile imine. Figure 4 shows the energy levels of the frontier orbitals for our set of compounds.The presence of electron withdrawing groups in the dipolarophile (Lewis acids attached to the N_2_ molecule) increases their electrophilicity and lowers the LUMO orbital in all cases, favouring the reaction. This is confirmed by the decrease of the barriers observed in most cases (Fig. 3). This effect is negligible only in the case of HF attached to N_2_, for which the gap is quite similar to the N_2_ system alone.

**Figure 5:**
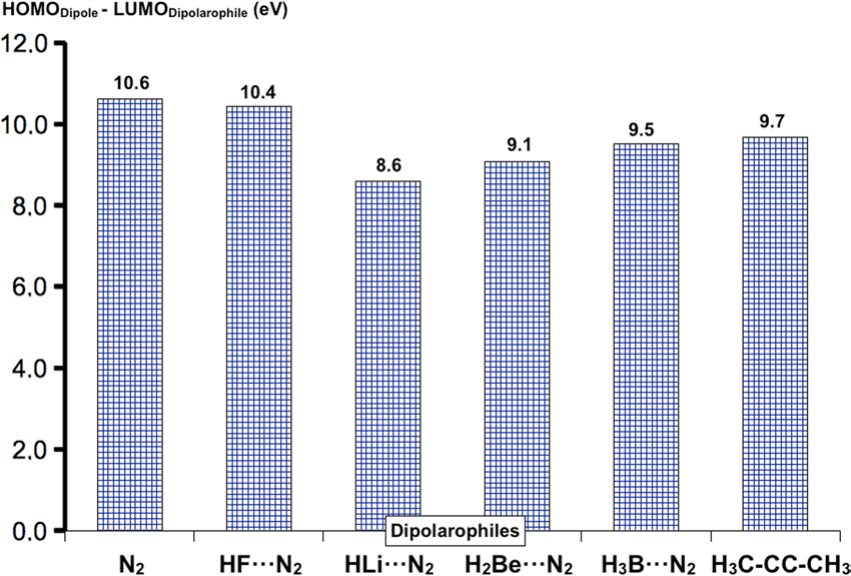
HOMO-LUMO gaps for the different neutral dipolarophiles (N_2_, FH···N_2_, HLi···N_2_, H_2_Be···N_2_, H_3_B···N_2_, H_3_CCCCH_3_) and the dipole (H_3_CNNCCH_3_) at the G4MP2 level of theory.

**Figure 6:**
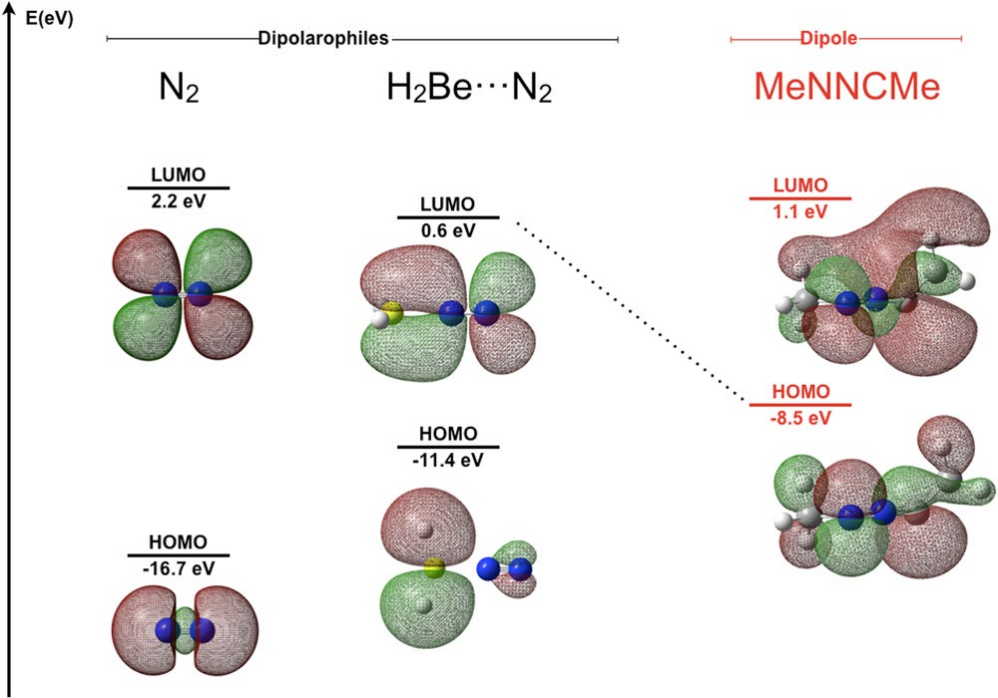
FMO model for the H_2_Be···N_2_+H_3_CNNCCH_3_ reaction at the G4MP2 level of theory. HOMO-LUMO levels for the N_2_ molecule are shown for the sake of comparison.

Figure 5 shows the HOMO (dipole) – LUMO (dipolarophile) gaps for the different neutral dipolarophiles considered. It might be noticed that although lower gaps are related to lower free energy barriers (see Table 1), this relation is not straightforward, as the FMO model in the ground state is used only as an approximation to the behaviour of the interacting system. However, the prediction is qualitatively good. Figure 6 illustrates the FMO model for the particular case of the H_2_Be···N_2_ + MeNNCMe reaction.

**Figure 7:**
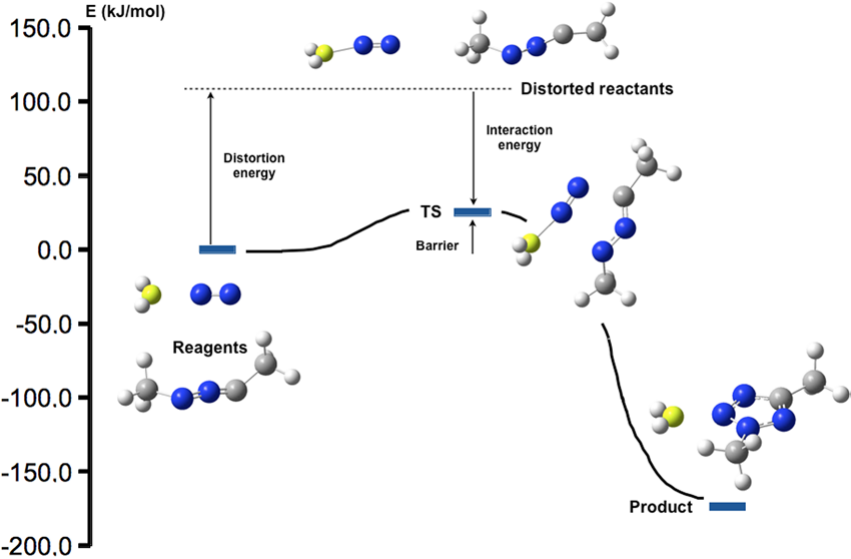
Energy profile for the reagents, transition state and products for the H_2_Be···N_2_ + H_3_CNNCCH_3_ reaction at the B3LYP/6-31 G(2fd,p) level of theory. The relationship between distortion energy, interaction energy and the barrier is also shown in the picture.

Finally, a distortion-interaction analysis was carried out at the transition states to evaluate the contributions to the catalytic effect observed for the different cases and for the beryllium hydride one in particular (see Fig. 7)^16^. According to G4MP2 calculations (Table 1), the catalytic effect due to the neutral molecule attached to N_2_ decreases in the order BeH_2_ > BH_3_ > LiH > FH, the latter one having almost no effect on the barrier. Within this approach, the energy barrier is decomposed into two main contributions, the distortion energy and the interaction energy. The distortion energy is the energy difference between the sum of the distorted reagents (at the geometry of the TS) and the optimized ones. The interaction energy is the energy difference between the TS and the fragments that are interacting, within the geometry of the TS. All in all, the energy barrier is the sum of these two contributions.

As single-point calculations at the G4MP2 level are not allowed in the G09 program used for the calculations^17^, the B3LYP/6-31G(2df,p) level was selected as the easiest way to have a qualitative description of the problem. This level is the one used in the first optimization cycle in the G4 method^18^. A first consequence is that the energy barriers, although in agreement with the range previously obtained with the accurate method, follow a slightly different order to the G4MP2 ones (see Table S1 in the Supporting Information). However, the most important conclusion is that the distortion energy is very similar in all the studied cases (including the non-catalysed reaction), the factor that makes the difference being the interaction energy. BeH_2_ in particular presents a very large value (−84.6 kJ·mol^–1^), remarkably larger than the one obtained for the non-catalysed reaction (−37.6 kJ·mol^–1^). This observation is in agreement with the previously reported ability of beryllium derivatives to strongly modify the properties of a given system. When beryllium acts as a Lewis acid, polarization effects can be so strong that there is a net charge transfer between the interacting moieties. The magnitude of these effects is related with changes on the intrinsic reactivity of both partners involved in the interaction^19–23^. Actually, Be is such a good acceptor towards N_2_ that the molecule presents an atomic charge difference of almost 0.2 a.u. upon interaction with beryllium hydride. The molecular nitrogen polarity is also significantly modified by BeH_2_ in the TS with respect to the non-catalysed reaction, as explained in detail in the NBO analysis results provided in the Supporting Information.

Finally, it is reasonable to think whether solvent effects or temperature could change the observed catalytic effects. With this purpose, we modelled through PCM^24^ calculations a highly polar environment as for instance water, which has been selected not as a realistic and suitable solvent for the reaction illustrated in Fig. 7, but just to analyse the effect on the energy barrier. Table 3 shows the comparison between the non-catalysed and the BeH_2_ catalysed reaction taking into account solvent effects and temperature. Although the transition state would be slightly destabilized with respect to the reagents in solution both for the non-catalysed and the catalysed cases, the catalytic effect in solution is still very large. Moreover, the products are also slightly stabilized with respect to the product. Regarding the effect of the temperature, we considered a value of 150 °C (423 K). Increasing the temperature gives place to even larger barriers for the catalysed and non-catalysed reactions (122.6 vs.163.9 kJ·mol^–1^), but again the difference between both values is similar to the one observed at 298 K.

**Table 3:** Vacuum versus water (T = 298 K, cursive; T = 423 K, brackets) enthalpy, free energy and entropy contributions (kJ.mol^–1^) for the 1,3-dipolar cycloaddition between dimethyl nitrile imine, and N_2_ and H_2_Be···N_2_ as dipolarophiles at the G4MP2 level (T = 298.15 K).

Tetrazoles	∆*H*	∆*G*	−T∆*S*
**Reagents: N**_**2**_	0.0	0.0	
2,5-Dimethyltetrazol (TS)	93.9	142.5	48.6
	*94.5*	*143.7*	*49.2*
	[93.5]	[163.9]	[69.4]
2,5-Dimethyltetrazol	−77.2	−24.9	52.3
	−*84.8*	−*32.0*	*52.8*
	[−*78.9*]	[−*2.2*]	[*76.7*]
**Reagents: N**_**2**_ **···BeH**_**2**_	0.0	0.0	
BeH_2_ (TS), Be close to N	42.5	98.9	56.4
	*47.5*	*106.2*	*58.7*
	[*42.3*]	[*122.6*]	[*80.3*]
BeH_2_ (TS), Be close to C	46.5	105.4	58.9
	*55.5*	*115.2*	*59.8*
	[*46.3*]	[*130.1*]	[*83.8*]
BeH_2_ linked to N3	−151.4	−90.0	61.4
	−*154.5*	−*92.7*	*61.8*
	[−*153.0*]	[−*65.4*]	[*87.6*]
BeH_2_ linked to N4	−171.7	−109.2	62.5
	−*180.7*	−*118.0*	*62.7*
	[−*173.3*]	[−*82.8*]	[*90.4*]

Dimethyl nitrile imine is omitted as a reagent for the sake of clarity.

In summary, accurate calculations at the G4MP2 level predict a huge decrease of the 1,3-dipolar cycloaddition reaction barrier between N_2_ and nitrile imines, by means of the activation of dinitrogen with Lewis acids such as BeH_2_ (>40 kJ·mol^–1^). The catalytic effect is the result of a sum of factors, among them a preference of BeH_2_ for tetrazole, a decrease of the energy level of the LUMO orbital, and a large interaction energy at the TS geometry, according to MESP, FMO theory and Distortion-Interaction Analysis, respectively. The main conclusion is that increasing the reactivity of N_2_ is not only possible with Fe (Haber-Bosch-Ertl process)^25^, with Fe-Mo (nitrogenases)^26^, or with nitrogenases and Cd^27^, or metal carbides^28^, but that neutral compounds containing boron or beryllium are able to activate dinitrogen, including possibly the corresponding metalloids or alkaline-earth metals.

## Methods

### Computational Details

Calculations were carried out by means of the Gaussian09 program^17^. All structures were fully optimized at the G4MP2 level of theory and followed by harmonic frequency calculations to characterize reagents, products and transition states^18^. G4MP2 barriers are in very good agreement with other accurate computational methods used for similar 1,3-dipolar cycloadditions^29,30^. In the Distortion-Interaction approach^16^, the energy barriers were recalculated at the B3LYP/6-31 G(2df,p) level of theory, the optimization level used in the G4MP2 composite method, to estimate the distortion and interaction contributions at the TS. The Self-Consistent Reaction Field (SCRF) methods consider the solvent as a continuum, which involves an implicit treatment of the solvent, characterized by a uniform dielectric constant. In the PCM (Polarized Continuum Model) in particular^23^, the solvent is embedded in a cavity constructed as a series of interlocking atomic spheres. The electrostatic potential of the solute interacts with the solvent creating an induced polarization, which has an electrostatic effect on the solute. Although explicit interactions cannot be taken into account in this method, it accounts for the bulk effects triggered by the solvent, which cannot be computed from explicit treatments. Natural Bond Orbital (NBO) method provides a decomposition of the molecular space that allows a Lewis picture of bonding in the system, defined in terms of core, bonding/anti-bonding and virtual orbitals, along with lone pair orbitals^31^. Also, the widely-used natural charges extracted from this analysis provide a clue about how the charge is distributed in the system.

## Electronic supplementary material

Supporting Information
