# Supplementary material for: Activation of Dinitrogen as A Dipolarophile in 1,3-Dipolar Cycloadditions: A Theoretical Study Using Nitrile Imines as “Octet” 1,3-Dipoles
Source: Sci Rep. 2017 Jul 21;7:6115. doi: 10.1038/s41598-017-05708-z (PMC5522451; doi:10.1038/s41598-017-05708-z)
Supplement: Supplementary file 1 — Supporting Information [file 41598_2017_5708_MOESM1_ESM.pdf]

SUPPORTING INFORMATION

Activation of Dinitrogen as Dipolarophile in 1,3-Dipolar Cycloadditions: A Theoretical Study Using Nitrile Imines as "Octet" 1,3-Dipoles

M. Merced Montero-Campillo,[†] Ibon Alkorta[†,*] and José Elguero[†]

[†]Instituto de Química Médica, CSIC, Juan de la Cierva, 3 , E-28006 Madrid, Spain
*E-mail: ibon@iqm.csic.es

Total number of pages: 4

Contents:
Page S2. Molecular Electrostatic Potential (MESP).
Page S3. Distortion-Interaction Analysis.
Page S4. Natural Bond Orbital (NBO) analysis.

Molecular Electrostatic Potential (MESP) results for the BeH2-catalyzed reaction.

The location of the MESP minima values (au) obtained with the DAMQT program[1] using the G4-MP2 wavefunction is shown in Figure S1 along with the corresponding values. These results indicate that a Lewis acid should bind tetrazole stronger (MESP minima of -0.071 and -0.091 au) than the TS (-0.054 and -0.036 au), being the complex formed with N_2 much weaker (-0.019 au). This partially explains the larger stabilization observed for the TS and tetrazole moieties.

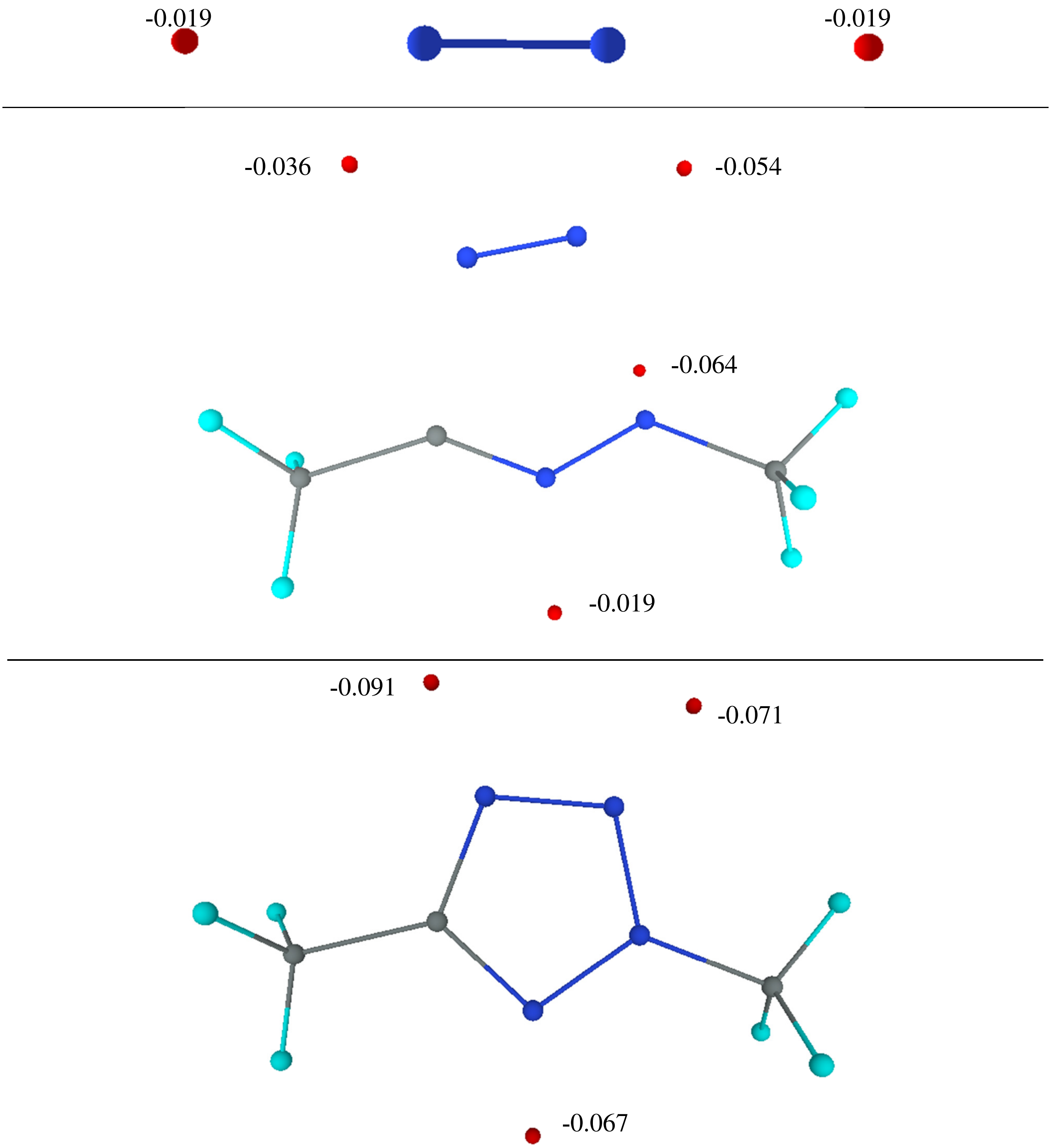

Figure S1. Location (red dots) and values (au) of the MESP minima.

Distortion-Interaction Analysis.

As explained in the text, within this approach, the energy barrier is decomposed into two main contributions, i.e, the distortion energy (E_{dist}) and the interaction energy (E_{int}).[2] The distortion energy is the energy difference between the sum of the distorted reagents (at the geometry of the TS) and the optimized ones. The interaction energy is the energy difference between the TS and the fragments that are interacting, within the geometry of the TS. The resulting energy barrier is then the sum of these two contributions (see Figure 7 in the paper).

Table S1. Distorsion and interaction energies at the B3LYP/6-31G(2df,p) level of theory.

Group attached to N_2	ΔE_{dist}	ΔE_{int}	$\Delta E^{\neq}$	$\Delta G^{\neq}$	$\Delta G^{\neq}$ (G4MP2)
None	118.2	-37.6	80.6	130.7	142.5
HF	121.2	-71.1	50.1	118.5	141.5
LiH	121.4	-101.4	20.1	81.4	107.5
BeH_2	109.9	-84.6	25.3	85.4	98.9
BH_3	126.5	-72.5	54.0	108.3	104.2

As shown in **Table S1**, BeH_2 reduces significantly the non-catalysed barrier, as well as LiH and BH_3. Looking at the different contributions in columns 2 (ΔE_{dist}) and 3 (ΔE_{int}), very similar values (around 120 kJ/mol) are obtained for the distortion contribution in all cases. Even more, this latter contribution is slightly smaller than the others for the beryllium case. The TS of the catalysed reactions present much larger interaction energies than the non-catalysed one.

References

[1] Kumar, A.; Yeole, S.D.; Gadre, S.R.; Lopez, R.; Rico, J.F.; Ramırez, G.; Ema, I.; Zorrilla, D. *J. Comput. Chem.* **2015**, *36*, 2350.
[2] Ess, D. H.; Houk, K. N. *J. Am. Chem. Soc.* **2007**, *129*, 10646.

Natural Bond Orbital (NBO) Analysis.[1]

The NBO decomposition scheme provides a traditional Lewis picture of molecular systems. In this sense, NBO allows explaining bonding in the molecule in terms of CR (core orbitals), BD (bonding orbitals), LP (lone pairs) and RY (Rydberg orbitals). Usually, non-covalent or weak interactions can also be analyzed by looking at the interaction energies between occupied orbitals from the donor and empty orbitals of the acceptor.

As explained in the article, we exemplified through the particular case of BeH_2 (the most efficient catalyst of the series) the observed catalytic effect. Usually, beryllium gives place to strong non-covalent interactions (beryllium bonds) in donor-acceptor pairs. In our case, the interaction between BeH_2 and N_2 in the reactant is so strong, that the NBO results do not simply show a donor-acceptor interaction. Actually, a BD between both entities (mostly belonging to nitrogen) appears, with a Be-N distance of 1.8 Å. Polarization effects triggered by beryllium hydride also result into a substantial difference of charge in the nitrogen moiety (around 0.2), as detailed in the picture below. A similar situation is found in the transition states, in which the Be-N distances are even shorter. The N_2 moiety is differently polarized in both TS with respect to the non-catalyzed TS (see Δq values in the picture).

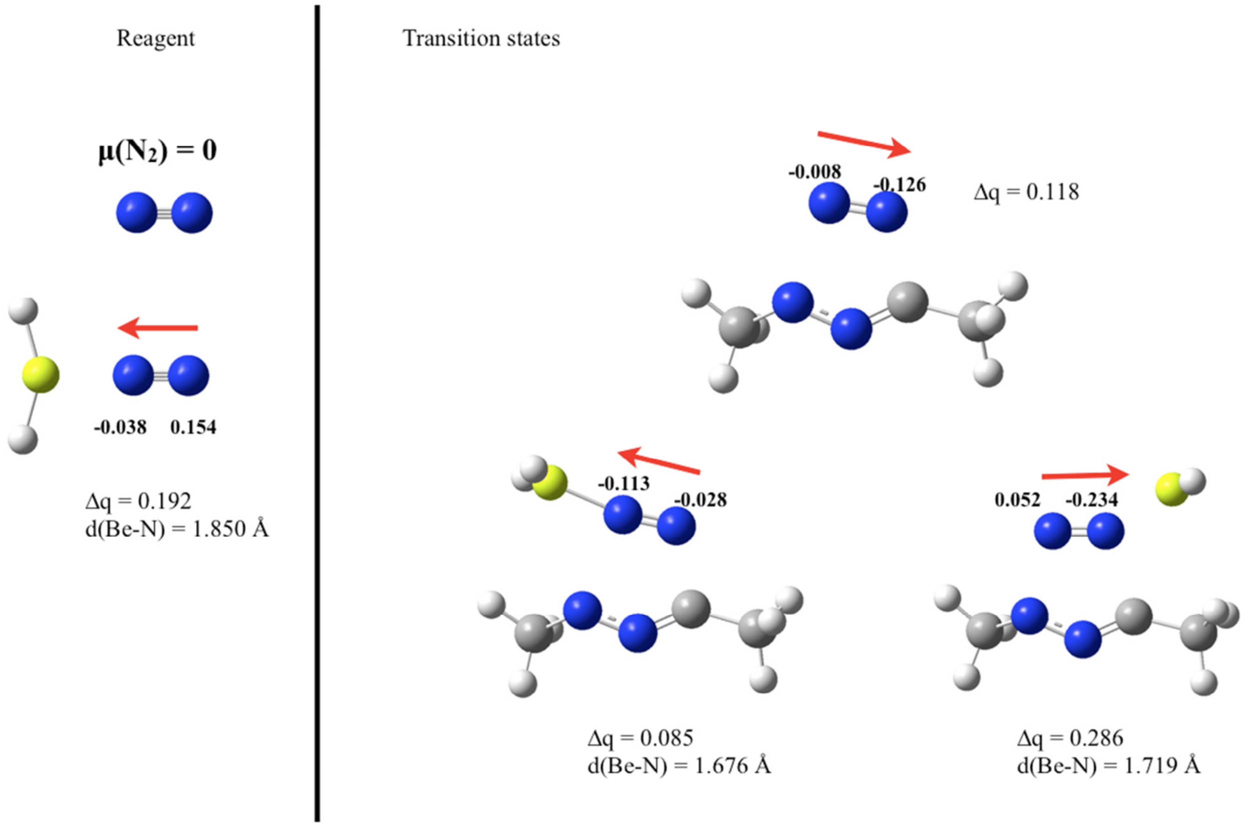

References

[1] NBO Version 3.1, E. D. Glendening, A. E. Reed, J. E. Carpenter, F. Weinhold.
